# C-Terminal Region of Caveolin-3 Contains a Stretch of Amino Acid Residues Capable of Diminishing Symptoms of Experimental Autoimmune Encephalomyelitis but Not Rheumatoid Arthritis Modeled in Rats

**DOI:** 10.3390/biomedicines11102855

**Published:** 2023-10-21

**Authors:** Alexey V. Danilkovich, Valery I. Turobov, Victor A. Palikov, Yulia A. Palikova, Anna O. Shepelyakovskaya, Evgeniy S. Mikhaylov, Gulsara A. Slashcheva, Tatiana E. Shadrina, Elvira R. Shaykhutdinova, Ekaterina A. Rasskazova, Elena A. Tukhovskaya, Oksana N. Khokhlova, Igor A. Dyachenko, Alina M. Ismailova, Dmitry V. Zinchenko, Elena V. Navolotskaya, Valery M. Lipkin, Arkady N. Murashev, Igor. P. Udovichenko

**Affiliations:** 1 State Center for Sciences by Shemyakin-Ovchinnikov Institute of Bioorganic Chemistry, Russian Academy of Sciences (BIBCh RAS), 6 Prospekt Nauki, 142290 Pushchino, Russiakhohlova@bibch.ru (O.N.K.); zdv@bibch.ru (D.V.Z.);; 2 Fundamental Biotechnology Department, RosBioTech University at Pushchino, 3 Prospekt Nauki, 142290 Pushchino, Russia

**Keywords:** encephalomyelitis (EAE), collagen-induced arthritis (CIA), peptide, autoimmune disease, drugs, caveolin, immunocortin, multiple sclerosis (MS), animal model, major histocompatibility complex (MHC), Dark Agouti (DA) rats, Sprague–Dawley (SD) rats

## Abstract

A short synthetic peptide from the C-terminal part of the caveolin-3 structure was tested for experimental autoimmune encephalomyelitis (EAE) treatment in rats. The structure–function similarity established between the novel synthetic peptide of pCav3 and the well-known immunomodulator immunocortin determined pCav3’s ability to reduce EAE symptoms in Dark Agouti (DA) rats injected with pCav3 (500 µg/kg). pCav3 was found to interfere with the proliferation of lymphocytes extracted from the LNs of DA rats primed with homogenate injection, with IC_50_ = 0.42 μM (2.35 mcg/mL). pCav3 affected EAE in a very similar manner as immunocortin. The high degree of homology between the amino acid sequences of pCav3 and immunocortin corresponded well with the therapeutic activities of both peptides, as demonstrated on EAE. The latter peptide, possessing a homologous structure to pCav3, was also tested on EAE to explore whether there were structural restrictions between these peptides implied by the MHC-involved cell machinery. Consequently, immunocortin was further examined with a different autoimmune disease model, collagen-induced arthritis (CIA), established in Sprague–Dawley rats. CIA was established using an intentionally different genetic platform than EAE. Based on the results, it was concluded that the effectiveness of pCav3 and immunocortin peptides in EAE rat model was almost identical, but differed in the rat model of rheumatoid arthritis; thus, efficacy may be sensitive to the MHC type of animals used to establish the autoimmune disease model.

## 1. Introduction

Multiple sclerosis (MS) is a severe, life-long neurological disorder of autoimmune origin with an unclear etiology. The communities most affected by multiple sclerosis are in industrialized countries, but in recent decades the disease has spread throughout the world. The availability of both improved diagnostic and medical services has influenced local statistics on MS patients. Since no data were available on MS prevalence in the human population during the SARS-2 pandemic, we operated with a previously reported level—149.2 cases per 100,000 individuals in 2012 in the USA [1]. Because MS is a life-long disease, MS countermeasures applied to patients pose a heavy financial burden on medical and insurance services; the annual average cost of disease-modifying therapies in the USA was reportedly up to USD 70,000 a year [2]. The detailed mechanism of MS onset is yet unknown, but it is continuously speculated that autoimmune developments in the immune system are linked to certain aspects of lifestyle, genetic predisposition, or the general ecological situation. Following the innate immune response, cells become capable of activating antigen-specific B and T cells. Such an event evinces the adaptive immune response in general [3,4]. The adaptive immune response contains both humoral and cellular parts, while the humoral response is fueled by B cells, the activation of which provides the organism with antibodies produced via circulating plasma cells. Generated antibodies can specifically target not only autologous antigens but also those with similar 3D structures. The function of antibodies is to provide antigen neutralization and opsonization, initiating the system of complementing and preventing pathogen proliferation [5]. B cell activation depends on the interaction between antigens loaded onto MHC and those antigen presenting cells (APCs) exposed to B cells. This process can be tuned by the magnitude of assistance provided by helper T cells (Th), which are CD+ type cells that promote the activation of CD8+ and CD4+ cells, while T cells continue to recognize particular antigens presented on the cell surface by MHC-I and MHC-II protein complexes [4]. As a result, T cells become capable of identifying infected cells and recruiting other immune cells to eliminate all detected damage [4]. Moreover, both B and T cells create limited populations of immune memory cells, which are capable of providing a faster adaptive immune response in case of reinfection with the same antigen. Obviously, a pool of antigen-specific receptors with extremely diverse specificity, located on the outer membranes of lymphocytes, makes the immune system capable of targeting the most pathogens. At the same time, even a power of such magnitude still possesses some sort of physical “Trojan” emerging from the nature of a universally wide range of antigen receptors. This could drive lymphocytes to acting specifically toward some antigen structures formally related to the native body and defined, therefore, as self-antigens. Normally, such lymphocytes capable of activating with self-antigens should be eliminated in the course of immune cell maturation. Indeed, there are mechanisms directed to control immune cell specificity to cell antigens, which aim to cancel auto-reactive lymphocyte propagation. At the same time, a tolerance mechanism has to be employed during lymphocyte activation to prevent the potentially auto-reactive lymphocytes from escaping negative selectionand to allow the eventual onset of autoimmune disease after they are exposed to unidentified antigenic stimuli. Similar mechanisms have been observed in multiple sclerosis (MS), type-1 diabetes (T1D), and rheumatoid arthritis (RA) [4].

Autoimmune diseases can be triggered by B and T cells, recognized as self-antigens, and then become activated to promote the proliferation of T cells with defined antigenic specificity. The key reason for neuron malfunctioning in patients diagnosed with MS is the impairment of myelin sheath integrity in the neurons in the central nervous system (CNS). Myelin, which insulates nerves, is composed of distinctive components—myelin basic protein (MBP), proteolipid protein (PLP), myelin-associated glycoprotein (MAG), and myelin oligodendrocyte glycoprotein (MOG) [6]. Demyelinization in MS has several clinical manifestations, grouped into four disease forms: relapsing-remitting MS (RRMS), which accounts for 85% of all registered MS cases; primary progressing MS (PPMS), which is diagnosed in 10% cases; progressive-remitting MS (PRMS), which relates to another 5% cases; and secondary progressive MS (SPMS), which usually develops over time in some patients previously diagnosed with RRMS [7]. Despite the precise switching of MS being unclear, both T and B lymphocytes are known for taking part in MS progression. While T cells are assumed to play the main role in the event, B cells are also capable of penetrating the hematoencephalic barrier by infiltrating both the parenchyma and cerebrospinal liquid. Finally, the infiltrated cells become responsible for propagating distinctive oligoclonal antibodies, which are joined to the myelin components with a high affinity [8]. Such B lymphocytes were characterized with an elevated expression of lympho-proliferative cytokine GM-CSF [9]. Loaded to MHC, class I antigens can promote the formation of peptide–MHC (pMHC) complexes, capable of directing the immune response and CD8+ cytotoxic T-cell (CTL) activation [10,11]. Obviously, the antigen structure restriction, achieved with MHC and TCR proteins, lowers the overall number of self-antigens among the pool of peptidome structures, since they are loading to MHC in a competitive manner. Thus, the volume of the T-cell receptor (TCR) repertoire influences the magnitude of the T-cell response, while naive T cells must provide recognition for every potential peptide generated within the body and complexed with MHC proteins in order to adjust the adaptive immune response [12,13]. For instance, EBV immune recognition is usually dominated by a single EBNA3 peptide bound to HLA-B8 [14]. HLA-B8-restricted T-cell response in humans is mainly conducted by a single TCR type (LC13), which is considered to be a public clonotype structure shared among all HLA-B8-expressed individuals [15]. A study of TCR immunodominance during the response to Epstein–Barr virus (EBV) has shown that such a structure of pMHC complexes may exist to affect the selection of immune-dominant peptides and disrupt the selection of the fittest structures. It is not clear what exactly distinguishes between the prospective ligand structures and less prominent candidates for this role. Presumably, the auto-immune process could be influenced by the antigens able to compete with the structures that promote pathology by forming an effective immune complex with distinctive MHC and TCR receptors. Pathology-inducing antigens are therefore defined as pathology-related factors.

There are several medications approved to treat MS symptoms in clinics: massive (several grams) intravenous injections of donor plasma immunoglobulins (IVIG) low-specific agents with cytostatic activity, which are also used to treat rheumatoid arthritis (RA), steroid immunosupressants. Historically, the most typical treatments of MS in humans are based on IFN β-1a, IFN β-1b, glatiramer acetate (Copaxone-1), and dimethyl fumarate [16,17]. Several immune-based anti-MS drugs have been approved for humans, including ocrelizumab (mAb to CD20 B-cell antigen) [18]; natalizumab (Tysabri^®^ by Vetter Farma-Fertigung, Germany; mAb to the α4 integrin) [19]; daclizumab (mAb to the subunit of IL-2Rα [20]); mitoxantrone (Novantrone^®^), which is a rather systemic cytostatic agent that also possesses tropism for immune cells [21]. Finally, fingolimod (Gilenya), known for interfering with the infiltration of lymphocytes into the central nervous system (CNS) by blocking the cell penetration of the blood–brain barrier, similar to the non-selective agonist of sphingosine-1-phosphate receptor FTY720 (S1P) activity [22].

Glatiramer acetate (Copaxone-1^®^ by Teva Pharmaceuticals) is the only polypeptide approved by the FDA as a drug for MS treatment. Copaxone is composed of four amino acid residues, found in the myelin most frequently. Glatiramer acetate is assumed to possess remarkable activity by interfering with myelin-directed autoantibody (auto-Ab) accumulation and cytotoxic CD8+ T-cell proliferation [6]. Therefore, Copaxone enables a very natural way of introducing glatiramer-derived peptides to the inner anti-gene restricting machinery in the immune system. Notably, this drug is greatly demanded on the market, given the remarkably small list of unfavorable side effects compared to other medications, despite the fact that the efficacy of modern MS treatments, on average, remains below 25%. Furthermore, the monoclonal antibodies and cytokines used for MS treatment often produce deep distress and promote immune system deficiency in MS patients. Immune-modifying drugs are noted for having lower efficacy against the primary and secondary non-remitting (progressive) forms of MS, which are less prevalent than RRMS but could exhibit highly severe symptoms [23]. To summarize, there is a constant need to search for novel immune-modifiers able to treat MS, especially its progressive forms.

Immunosuppressive peptide structures with anti-proliferative activity toward activated lymphocytes emerged several decades ago. Since then, some endogenous peptides of humans were determined to possess certain immunosuppressive activities, lacking most of the side effects usually observed during MS treatment with common medications. The prime structure of immunocortins is composed of ValLysLysProGlySerSerValLysVal amino acid residues (a.a.). This sequence was derived from immunoglobulin. It is homologous to the N-terminal region of human Ig γ- or μ- heavy chains (from 11 to 20 a.a.), just upstream to the variable VH-region [24]. Although immunocortin is known for exhibiting immunosuppressing properties, its practical implications remain under consideration.

During this work, we studied a short synthetic peptide (pCav3) found in the C-terminal region of caveolin-3, chosen because its primary structure was found to be similar to that of immunocortin. This novel peptide structure is located at the C-terminus of the primary human caveolin-3 structure. The main properties of pCav3 were evaluated by emulating a 3D pCav3 model and then placed in a triple auto-immune complex MHC/TCR/p (SwissProt ID 2Z31) by replacing the peptide ligand of the myelin origin in silico. Given the molecular docking results, pCav3 was picked up to test its activity in vitro and in vivo. We decided it would be rational to compare the same peptide treatments on two models of different autoimmune disorders. This could provide additional information on the antigenic peptide structure influenced by the treatment specificities. We used experimental autoimmune encephalomyelitis (EAE) as the MS model and collagen-induced arthritis (CIA) as the model of rheumatoid arthritis (RA). Both models went through the same peptide treatments. Similar to EAE, the CIA used has been established in several rat strains, including Sprague–Dawley (SD) immunized with collagen type II [25,26]. Also, CIA was shown to share both immunological and pathological features common to rheumatoid arthritis in humans (RA); so far, it has been used to study RA [27]. Contrary to the EAE that was optimized for DA rats, the CIA model was established on different from DA immunogenetics corresponding to distinct rat lineages susceptible to CIA. This point makes a bridge between the features of the autoimmune disease model and the antigenic specificity of the MHC proteins in the animals used for modeling the disease [28,29]. It is known that rat strains with different MHC types are able to develop CIA, although the autoantibody specificities or T-cell epitope affinities may differ among the rat strains significantly [30]. For instance, DA and SD rats have distinct MHC RT1 haplotypes av1 and f respectively [25,29]. Although DA rats are highly susceptible to immunization with collagen II, these animals are known to employ two different immune mechanisms that are engaged simultaneously during the immune system response to collagen II in the course of CIA. Therefore, DA rats are explicitly not recommended by the Organization of Economic Cooperation and Development in Europe (OECD) to model CIA. This point is highly questionable from the authors’ prospective.

The aims of this study were to investigate the novel peptide pCav3 homologous to the immunocortin structure for its potency in treating an autoimmune model of EAE, while immunocortin was examined for the treatment of EAE and CIA established in DA and SD rats, respectively. These two models both relate to autoimmune disorders in humans, but were induced with distinct antigens applied to the different genetic landscapes featured by the DA and SD rats. This comprehensive approach makes enabled the investigation of immunosuppressive peptide activity by monitoring the magnitude of peptide-directed effects on certain autoimmune diseases to clarify the prospects of autoimmune disease treatments in clinics.

## 2. Materials and Methods

### 2.1. Peptide and Structure Analysis

Search for the regions of homology to immunocortin was accomplished by analysis of the protein database GenProt at https://ncbi.nlm.nih.gov (accessed on 10 February 2023), and then implementing them using the BLAST website. Eventually, the structure of interest was found in the C-terminal region of human protein caveolin-3 (Cav-3). A 3D structure of peptide pCav3 was modeled and processed using SPDB viewer v.3 software. A triple complex (SwissProt ID 2Z31) of myelin peptide, complexed with TCR/MHC proteins (MHC/pep/TCR), was used as a template in silico replacement of the ligand in 2Z31 with the investigated structure. Free energies of the isolated ligand and receptor proteins MHC/TCR or those of the whole complex were assessed with a specialized subroutine of the software. The estimated values were used to determine the ability of the ligands to bind to the corresponding receptors. The free energy of binding (*E_bind_*) were calculated using the framework of the generalized approach by Born, since *E_MM/GBSA_* (the free energy of the structure) include solvation effects, taking into account the size and kind of the molecule surface exposed to the solution [31]:

Δ*E_MM/GBSA_* = Δ*E_MM_* + Δ*E_GBSA_*

where Δ*E_MM_*—molecular mechanic energy; Δ*E_GB_*—free energy presented by the generalized Born approach, including mechanical, electrostatic, and Van der Vaals interactions; Δ*E_SA_*—events due to molecular surface solvation (SA). The free energy of ligands binding to the complex of TCR and MHC proteins was calculated accordingly:

*E_bind_* = Δ*E_complex_* − (Δ*E_ligand_* + Δ*E_TCR_/_MHC_*),

where Δ*E_complex_*—free energy of the full complex; Δ*E_pep_*—free energy of the isolated ligand; Δ*E_TCR_/_MHC_*—free energy of the “empty” complex (structure without ligand). Given the ligand affinity was confirmed in silico, the chemical synthesis of pCav3 peptides and immunocortin was accomplished. The peptides were crafted with a standard solid-phase peptide synthesis (SPPS) approach using Fmoc/tBu-protected amino acids as precursors [32,33]. The peptides were purified from the main preparation mixture with reversed-phase chromatography and a UV-light detector set at 226 nm (2238 Uvicord SII, LKB Bromma). The peptide homogeneity was confirmed with a specific HPLC analysis (>95.5% homogeneity), which determined it was suitable for biological trials and preclinical study.

### 2.2. Animals

The experimental protocols used in this work fully complied with the International Guiding Principles for Biomedical Research Involving Animals issued by WHO. The experiments were approved by the Institutional Commission for Control and Use of Laboratory Animals at the Branch of the Shemyakin-Ovchinnikov Institute of Bioorganic Chemistry, Russian Academy of Sciences (protocol number: 711/19 on 23 September 2019). The study was conducted at the Animal Care Facility of the Laboratory of Biological Trials at BIBCH RAS (Pushchino, Russia). The animal care procedures were performed according to the laboratory SOPs. The room that was inhabited with the animals in the experiment has a certified barrier to external media. There was an automatic period of switching between day and night every 12 h, and the whole volume of circulating air changed every 1 h. The temperature and humidity were monitored in each room remotely with the Eksis Visual Lab computerized system, Practice-NC Ltd., Moscow, Russia. Dark Agouti (DA) rats (DA/ZFV Crl BR breeding stock) were purchased from Charles River Co. (Sulzfeld, Germany). DA and SD rats were bred at the Shemyakin and Ovchinnikov Institute of Bioorganic Chemistry, Pushchino, Russia. Outbred female DA rats that were 10–12 weeks old and weighed 190–220 g were used in the experiment. Outbred SPF female SD rats that were 8–12 week old and weighed 170–200 g were used in the experiment after they grew at the breeding facility “Pushchino” at the Shemyakin and Ovchinnikov Institute of Bioorganic Chemistry, Russian Academy of Sciences (Pushchino, Russia). The animals were kept at the animal facility under climate-controlled conditions with 12 h light/dark cycles, fed food, and provided with water ad libitum. All manipulations applied to the animals were evaluated and approved by the Institutional Animal Care and Use Committees (IACUC, Singapore). The animals were accommodated for 2 weeks before the experiment started; they were kept in secure isolated rooms under controlled environmental conditions: temperature of 20–24 °C; humidity of 30–60%; 12 h light/dark cycle. The animals were fed ad libitum (SSNIFF V1534-300, Spezialdiaeten, GmbH, Stuttgart, Germany) and had free access to Milli-RO (Millipore, Burlington, MA, USA) purified sink water. The cages were supplied with an ecologically pure Lignocel Nesting Ball (JRS, Rosenberg, Germany) and pyramids of red polycarbonate for the animals to entertain themselves during their inhabitation period (Tecniplast S.P.A., Varese, Italy). The study was conducted fully in accordance with the following regulating documents: the Institutional Animal Care Rules and National User Program, Federal Guidelines SP 2.2.1.3218-14; Guide for the Care and Use of Laboratory Animals: Eighth Edition (National Research Council, Washington, DC, USA, 2011), Directive 2010/63/EU of EP and the National Research Council on 09.22.2010 regarding animal protection during scientific manipulations; Guidelines by the National Research Council, 2003 (Care and Use of Mammals in Neuroscience and Behavioral Research).

### 2.3. Experimental Autoimmune Encephalomyelitis Model (EAE)

DA rats weighing 210–230 g were divided into three groups with six animals in each. Homogenate injection for DA rat immunization was prepared by homogenizing spinal cords that were previously excised from rats, frozen, and stored in liquid nitrogen until use. During the first step of immunization, the homogenate injection was emulsified with incomplete Freund’s adjuvant (IFA, 1:1 *w*/*v*), as described elsewhere [34]. Rats were immunized with 50 μL of the syngeneic homogenate drug injected into both hind footpads. The animals’ condition and weight were monitored for EAE clinical symptoms according to the following clinical scores: 0, asymptomatic; 1, loss of tail turgor; 2, impaired righting reflex; 3, partial paralysis; 4, complete paralysis; 5, moribund or dead animal. The clinical signs of sub-typical symptoms were scored as 0.5 less than the symptoms graded above. In our case, acute-phase EAE in the DA rats started in the control group of animals with non-treated EAE on the 12th day after immunization with homogenate injection and continued until day 14, which corresponded to the most obvious signs of the disease detected in the experiment. The peak of disease symptoms (acute phase) lasted for 3 days and were characterized by the biggest scores detected in the EAE animals in the experimental groups. Overall, EAE was scored once daily in each group of animals used for the experiment. The common cumulative scores of groups were calculated once daily during three phases of EAE: developing (shown on day 1 only); acute phase (from day 12 to 14); recovering (from day 15 to 19). Such an approach was used to analyze the data on the EAE phenotypes obtained during the experiment. The annual data on the cumulative scores were confirmed with the Mann–Whitney (U-test) criteria, Kholmogorov–Smirnov, and Kruskal–Wallis tests at *p* < 0.05. The animals were euthanized at the end of the experiment in a CO_2_ chamber.

### 2.4. Peptide Therapy for EAE

Starting the day after EAE was induced with immunization of DA rats by homogenate injection, the animals of the experimental groups were injected intraperitoneally (i.p.) with a tested peptide solution (100 μL) at a dose of 500 mcg/kg once a day. The control group of animals received a placebo-equal volume (100 μL) of a carrier-solution of non-pyrogenic saline (0.9% NaCl). The experimental groups of animals, which received the tested peptides, were considered the “treated EAE” groups. The control group of animals, which received the carrier only, was considered the “non-treated EAE” group. The rats in the experimental groups took the treatment with pCav3 or immunocortin once a day, while the control animals took the placebo (carrier). The EAE scores were estimated for every animal starting on day 11. The individual scores in each group were used to calculate a combined integral score of the group each day until day 18. Starting on day 19, all of the animals were considered fully recovered, and the peptide injections were discontinued. At this point, the whole experiment was considered to be over.

### 2.5. Histological Examination

Histological EAE analysis was performed on sections made from the lumbar spinal cords of the EAE-treated and non-treated DA rats prepared on day 12 after EAE initiation. The rats were euthanized, and then each rat was perfused with 4% paraformaldehyde in 0.1 M phosphate buffer. The spinal cord was carefully removed and then immersed in the same fixative. The specimens’ spinal cord lumbar segments were embedded in paraffin, and 5 μm-thick slices were cut with a microtome to be stained and analyzed.

The slices were stained with a standard hematoxylin–eosin (H&E) procedure and visualized using the AxioScope A1 (Carl Zeiss) microscope. Pictures were taken with the Axiocam 305 color camera (Carl Zeiss).

### 2.6. Collagen-Induced Arthritis (CIA) Model

A total of 40 female rats SD, including the control group (*n* = 8), were taken for the experiment. CIA was modeled by injecting collagen type II. Bovine collagen II was isolated and purified to yield powder in the Pharmkinetics Laboratory at IBCH RAS (Pushchino, Russia). The collagen was dissolved in 0.1 M acetic acid up to 2.5 mg/mL. For the immunization, the collagen solution was mixed with incomplete Freund adjuvant (IFA) at 1:1 ratio (*w*/*v*). The prepared emulsion contained collagen II at a concentration of 1.25 mg/mL. The emulsion of 50 μL of collagen II was injected into four places in each animal: twice intradermally to the tail and twice subcutaneously to the back near the tail core. The injections were repeated on the 7th day. The animals in the control group were injected in the same places with the same volumes of sterile saline (0.9% NaCl). CIA was monitored once a day during the experiment for body mass, paw diameter, and clinical signs of manifestation. The paws of the experimental animals were scored as follows:

0—Absence of erythema;

1—Mild redness and ankle or wrist swelling;

2—Moderate redness and swelling of ankle or wrist;

3—Severe redness and the entire paw swelling, including digits;

4—Heavily inflamed limb, with multiple joints involved.

On day 28, the rats that scored above grade 3 were selected and divided into two groups of eight animals, which were subdued for CIA healing either with the tested peptide or saline. CIA treatment with the peptide started on the 4th week with 0.5 mg/mL of the tested peptide solution in sterile saline. The peptide solution was injected once a day at a dose of 500 mcg/animal from the 4th to the 8th week. The paw diameters were measured at weeks 0, 2, 4, 6, and 8 of the study using a digital caliper. The data were used for scoring the clinical signs of CIA in the SD rats.

### 2.7. Disability Test of Animals with CIA

Static pressure was delivered to the animal paws at approximately 0, 2, 4, 6, and 8 weeks (since the study started) with an SWB TOUCH measuring device (Bioseb, Vitrolles, France). The data represent the measurable degree of disability in the animals with CIA with their weight distributed between their injured front paws supported by external bars and injured hind paws placed on the weight detector tabs of the measuring plate. Timely data from each detector were assessed independently as they were displayed on the LCD. The program unit allowed for calculating the average weight put on each paw for the period of time of monitoring.

### 2.8. DLN Mononuclear Cells

Drained lymph node lymphocytes (DLN mononuclear cells) were extracted from lymph nodes excised from the DA rats on the 7th day after the animals were immunized with homogenate injection emulsified in incomplete Freund adjuvant (IFA, 1:1 *w*/*v*). DLN were homogenized with a standard cell homogenizer. Cells with debris were loaded over a Ficoll-formed gradient at room temperature (d = 1.077 g/mL, BioloT, St. Petersburg, Russia) and centrifuged at 400× *g* for 35 min. DLN lymphocytes were collected from the corresponding zone in the Ficoll gradient, transferred to a fresh tube with Hanks solution, and then centrifuged at 400 g for 10 min. The procedure of cell washing with Hanks solution was repeated twice. Viable cells were counted with Trypan blue with the use of simple light microscopy (Mg × 400). DLN lymphocytes were cultivated in complete DMEM medium with 2 mM-glutamine, 2 mM Na^+^-pyruvate, 20 mM β-ME, 40 mcg/mL gentamycin, and 10% fetal calf serum (HyClone, Logan, Utah). The media and chemicals for cell culturing were purchased from Sigma-Aldrich (St. Louis, MO, USA).

### 2.9. Flow Cytometry

pCav3 peptide can affect the fractions of cells at the G1, G2 + M, and S phases of the cell cycle, which results in characteristic changes of the data on lymphocyte proliferation. The DNA quantity in the cells was studied with the Accui 3C (Japan) cytofluorimeter. DLN leukocytes, extracted from the DLN of the DA rats (5 × 10^6^ cells/), were stimulated either with ConA (5 µg/mL) or ConA+pCav3 (10 mcg/mL) and then incubated in a 5% CO_2_ atmosphere for 48 h at 37 °C. The DNA was stained with vital dye SYBR-GREEN I (1:10,000 dilution) before it was assessed in the cell population with the Accui 3C. The data on the DLN lymphocytes distributed by the DNA per cell load were used to compare the DNA/cell numbers after exposure to pCav3 peptide or to the carrier. The Accuri 3C cytofluorimeter (Japan) was used to measure DNA fluorescence for DLN lymphocyte of at least >3 × 10^6^ per sample. The data were used to locate the cells at the G1, S, and G2 + M stages of the cell cycle (CCP) to determine the cell number for the M2 fraction, which included cells with fluorescence (FL2-A) above 2,100,000 per cell. Further analysis was conducted according to the Accuri 3C manufacturer’s recommendations for preinstalled subroutines.

### 2.10. Lymphocyte Proliferation by MTT Test

The potency of the studied peptide to inhibit lymphocyte proliferation was assessed by an MTT test. DLN leukocytes from the DA rats immunized with homogenate injection were seeded at 2.5 × 10^5^ cells/mL (5 × 10^4^ cells/well) in a 96-well plate and stimulated with ConA (5 mcg/mL) to be further exposed to different concentrations of the testing peptide for 48 h. Th cell number per well was assessed with MTT reagent, which could develop a colored derivative inside the living cells only. The optical density of each well was measured with a plate reader at 550 nm. MTT protocol with some modifications was used [35]. The peptide to be tested was dissolved in cell media with 1% DMSO. Then, 10 μL of the tested peptide solution (1 μg/μL) was added to the experimental well containing 50,000 cells/200 μL. Two-fold serial dilutions were prepared using 100 μL of media with peptide transferred to a fresh well preloaded with 25,000 cells/100 μL and located in the next row on the plate. Plain media (100 μL) was used to fill up the well volume to 200 μL. Finally, the highest plate peptide concentration was 25 μg/mL, which decreased by half in each subsequent row of wells. The plates were incubated in a humidified incubator at 37 °C, 5% CO_2_ for 48 h. Then, 15 μL of MTT solution 5mg/mL was added to every well, and then the plate was returned to the incubator for another 3 h. The plates were centrifuged at 3000× *g* rpm (850 g) for 30 min at 6 °C in a refrigerated centrifuge (Sigma, Ronkonkoma, NY, USA). Supernatants were aspirated from the wells and 100 μL of DMSO was added to each well. The plates were slowly rotated on an orbital shaker at 50 rpm at room temperature for 5 min. The optical density of the wells was measured at 550 nm with the SpectraCount plate reader (Packard, Detroit, MI, USA). The ratio of the sample OD_550_ to the control (untreated cells) was calculated and presented as a percentage of the control. The data reflect the cell proliferation rate per well. The peptide concentrations were performed in triplicate, and the experiment was repeated several times.

### 2.11. Statistical Analysis

The data were analyzed with Microsoft Excel (descriptive statistics) and Statistica v.7.1 for Windows. The data on EAE were confirmed with the Kruskal–Wallis and Kholmogorov–Smirnov tests at *p* < 0.05 and then further analyzed with one-way ANOVA (HSD) at *p* < 0.05. A post hoc Tukey’s honestly significant difference procedure performed pairwise comparisons using the ANOVA data set. The F statistic accounted for the overall differences among the sample (n) means (Mn). Tukey’s HSD test determined if a significant difference between the various pairs of means was possible at the chosen significant level. The data on the CIA model were analyzed for two sexes separately. The repeated results were analyzed with a single-factor dispersion analysis (ANOVA). The quantitative results of the disability test were evaluated with non-parameter Mann–Whitney criteria (U-test). Illustrations were produced with GraphPad «Prism v.5 and Prism v.5.04» software. The data values displayed mean ± SEM values; the volume of each group of animals used in the EAE experiment was *n* = 6; the volume of each group used in the CIA experiment was *n* = 8.

## 3. Results

### 3.1. Peptide Modeling and Ligand Analysis

**Figure 1:**
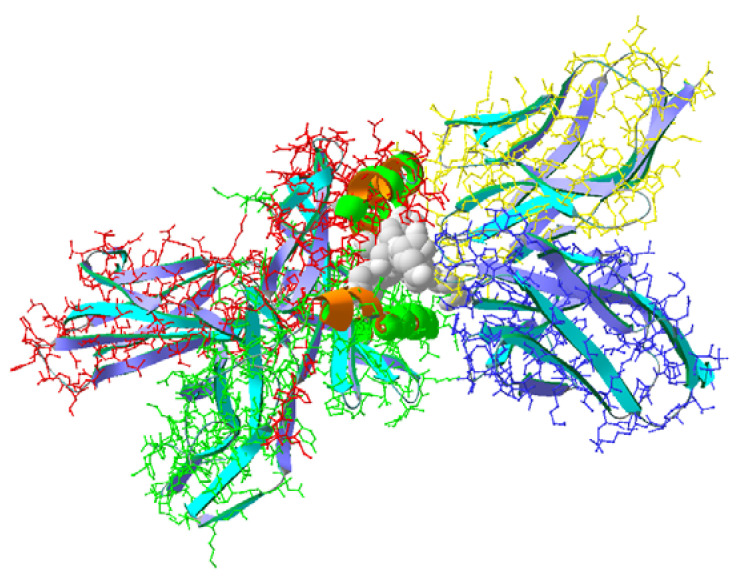
Triple complex 2Z31 topology; BMP peptide ligand (gray) complexed with heterodimers of MHC (red-green) and TCR (blue-yellow) proteins.

Three-dimensional models of isolated peptides were built with Swiss viewer v.4 (SPDV) software. X-ray data on the triple complex of myelin basic protein peptide ligand loaded to the TCR and MHC receptors (SwissProt ID 2Z31) were used for building similar complexes in which the ligands were replaced with the tested peptide structures (Figure 1). The new structures were minimized, and the ligand binding energies were calculated (Table 1). The calculated binding energy values indicated that the MBP ligand in 2Z31, comprising the MBP fragment, had a binding energy value of 403 kJ/mol, compared to pCav3 and immunocortin (506 and 496 kJ/mol, respectively). The calculated values of the binding energy for pCav3 and the immunocortin peptides are highly likely due to strong primary structure similarities observed at the C-termini of pCav3 and the immunocortin peptides (Table 1).

### 3.2. Peptide Effect on DLN Lymphocytes

**Figure 2:**
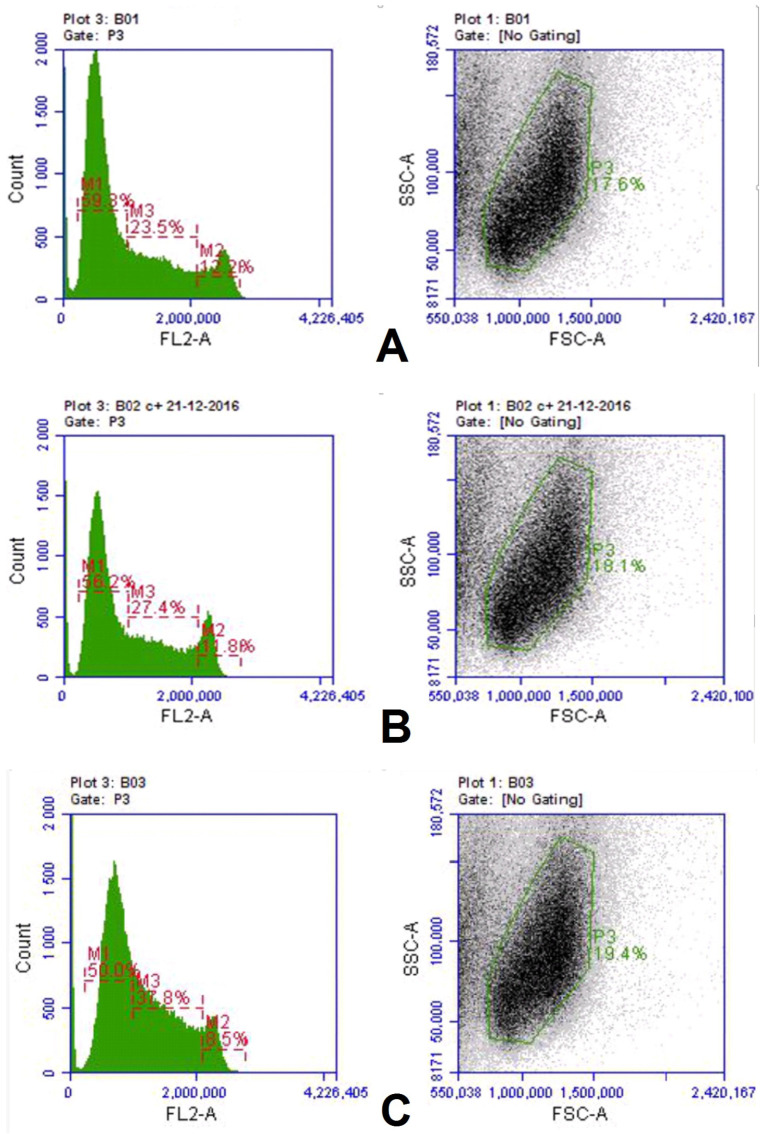
Peptide effects on cell cycle phase: cells were assessed by measuring DNA per cell content in M2 lymphocyte subpopulation (FL2-A > 2,100,000). Cytofluorimetric data on DLN lymphocytes of DA rats treated in vitro: (**A**) pCav3—20 mcg/mL; (**B**) pCav3—20 mcg/mL + ConA 5 mg/mL; (**C**) ConA 5 mg/mL. Axes are: X—fluorescence intensity; Y—fluorescence event (count). Response from untreated cells was statistically indistinguishable from cells treated with pCav3, and therefore, is not displayed.

**Figure 3:**
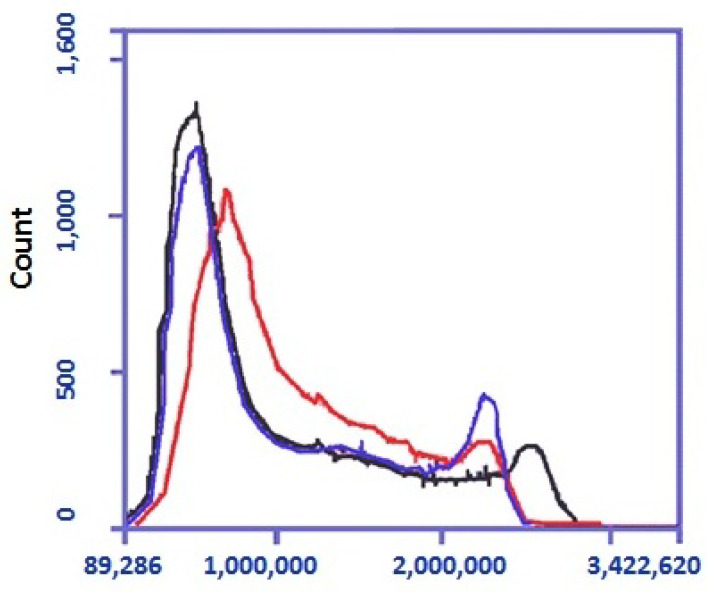
Distribution of DLN lymphocytes stained wth SYBER GREEN I. DLN lymphocytes, extracted from homogenate-primed DA rats on the 7th day, were treated in vitro with ConA—5 mg/mL (red); pCav3—20 mcg/mL + ConA 5 mg/mL (blue); pCav3—20 mcg/mL (black). Axes are: X—fluorescence intensity, Y—fluorescence event (count).

pCav3 was found to be able to affect the DNA content in the cells and the timing of the cell cycle (CC) phases. The DNA in the cells was stained with SYBER GREEN I, and then the cells were visualized with a cytofluorimeter (Accuri 3C, Japan) set to measure the DNA with a subroutine preinstalled by the manufacturer. DLN lymphocytes, plated in proper plates, were treated either with pCav3 + ConA or each component separately. The cells were stained with SYBER GREEN I. DNA loaded in the M2 subpopulations of cells was measured as having a higher FLA-2 fluorescence response (Figure 2). At least 100,000 cells of each sample were counted. An identical mask of the cytofluorimeter was applied to each sample to restrict viable and undamaged cells (Figure 2A–C). Data analysis on the DNA-containing cells revealed the cells treated with pCav3 at 20 mcg/mL + ConA 5 mcg/mL, yielding a large M2 lymphocyte population, as the cells had a higher DNA load per cell (FLA-2 > 2,100,000) (Figure 3). Some distinct changes are shown, as observed in the M2 subpopulation of the DLN lymphocytes as a result of different treatments: the G2/M phase of the cell cycle can be distinguished by the DNA quantity per cell because it is characterized by more DNA contained in the cell relative to the average of that in diploid cells (2n). The DNA per cell quantity was altered as a result of the treatment of DLN lymphocytes with pCav3 + ConA (Table 2). Obviously, the DLN lymphocytes treated with pCav3 peptide in the presence of ConA demonstrated affected cell proliferation (Figure 3). The main effect was related to a bigger subpopulation of premitotic cells compared to the cells treated with ConA 5 mg/mL only (Figure 3, second peak). The data in Table 2 summarize the effects of the different cell treatments. These results establish a valuable prerequisite for further analysis of the peptide activity both in vitro and in vivo. The rational prerequisites to consider are the symptoms developed in the course of auto-immune disease as related to the activation and proliferation of antigen-specific lymphocytes.

**Figure 4:**
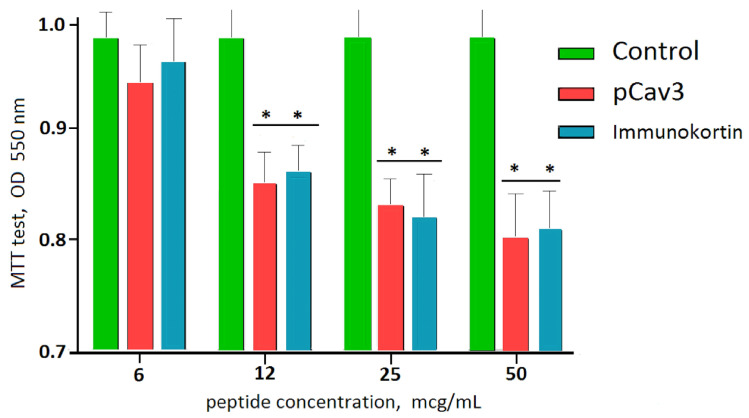
pCav-3 and immunocortin-influenced proliferation of peptide concentration >6 mcg/mL according to standard MTT test (OD 550 nm). Displayed are mean values and SEM, indicated with bars. Control represented by data on untreated cell proliferation assessed with MTT test. * Difference between proliferation of DLN lymphocytes in presence of pCav3 or immunocortin peptides and control (cells without peptide) were confirmed with Kruskal–Wallis and Kholmogorov–Smirnov tests (*p* < 0.05); difference between pCav3 and immunocortin influence on DLN lymphocyte proliferation was considered insignificant according to Kruskal–Wallis (*p* < 0.05, Kholmogorov–Smirnov tests (*p* < 0.05) and one-way ANOVA HSD (*p* < 0.05).

The biological effects of pCav3 and immunocortin are fairly similar given the significant similarity of the peptide structures. Both peptides were found to be able to suppress DLN lymphocyte proliferation in mixed culture. A greater inhibitory effect on cell proliferation in vitro corresponded to a peptide concentration of 12 mcg/mL or more (Figure 4). Given the sequence homologies between the pCav3 and the primary immunocortin structures (Table 1), both peptides demonstrated fairly similar biological effects in vitro.

### 3.3. EAE Peptide Treatments

**Figure 5:**
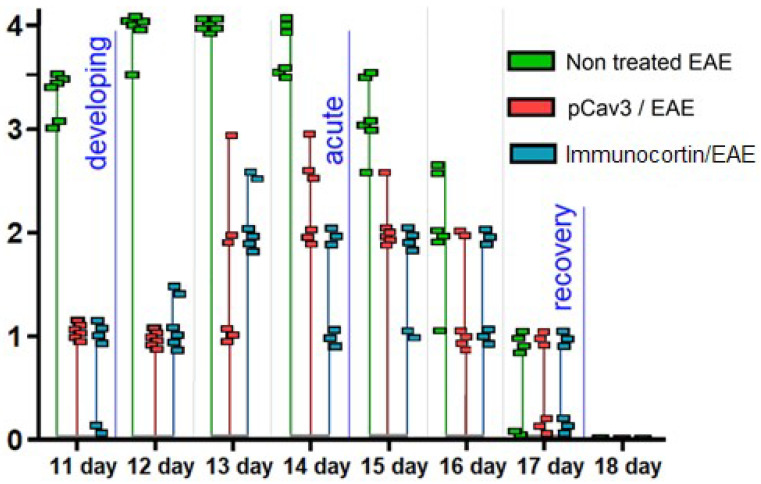
Peptides affected EAE during each phase (blue marked) of disease. Rats induced with EAE were injected once a day (500 mcg/kg) with pCav3 or immunocortin from days 2 to 18 of the experiment. Individual scores for DA rats in corresponding groups are displayed, starting on the 11th day after EAE was induced until disease symptoms vanished or were cured.

The data on the pCav3 activity on DLN lymphocyte proliferation laid the foundation for testing peptide activity in vivo using an EAE model in DA rats. A standard MS model in DA rats was induced with a single injection of syngeneic homogenate containing myelin-type antigens. In 10 days after the homogenate was injected, the experimental autoimmune encephalomyelitis (EAE) was developed in DA rats. The data on EAE treated with pCav3 or immunocortin peptides are displayed in Figure 5 from the 11th day since EAE was induced. It can be seen that the acute phase symptoms of EAE lasted for three days, from day 12 to 14, in the animals of the control group and from day 13 to 15 in the pCav3 or immunocortin-treated animals with EAE since the date the homogenate was injected. The EAE scores of the animals treated with pCav3 or immunocortin peptides did not reach the scores of the animals with non-treated EAE (Table 3), yet the activity of the pCav3 and immunocortin on EAE are indistinguishable according to the data on the EAE peptide treatment, evaluated using one way ANOVA (Table 3).

Pairwise comparisons within the ANOVA data were additionally assessed with Tukey’s honestly significant difference (HSD) procedure. Comparison of the control and pCav3 groups for the means of M_1_ = 15.81 and M_2_ = 7.44 yielded HSD = 8.38, while comparison of the control and immunocortin groups for the means of M_1_ = 15.81 and M_2_ = 6.88 corresponded to HSD = 8.94. Given HSD_.05_ = 8.1121, both cases of the pCav3 and immunocortin treatments differed from the control significantly with *p* = 0.04223 and *p* = 0.02918, respectively. At the same time, comparison between the pCav3 and immunocortin treatments resulted in HSD = 0.56, which corresponded to *p* = 0.98331, and therefore, the difference between these treatments was statistically insignificant.

### 3.4. H&E Staining and Analysis

**Figure 6:**
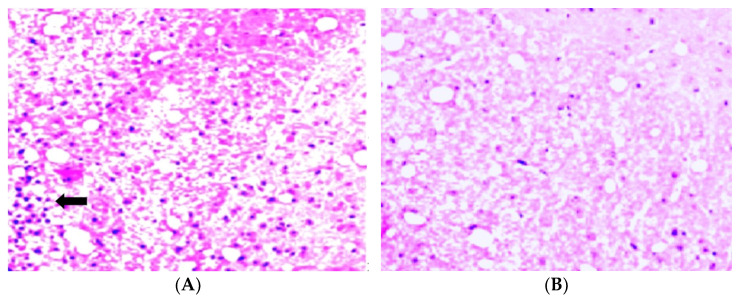
Hematoxylin–eosin staining (H&E) of rat lumbalic spinal cord. (**A**) EAE, non-treated. In the left bottom corner, congestion of infiltrated inflammatory cells (cyan) (arrow), mag. ×400; (**B**) EAE treated with a single daily injection of pCav3 (500 mcg/kg) for 1 to 18 days, spinal cord was taken on day 12 after DA rats were immunized with homogenate injection.

Standard H&E staining was used to illustrate the peptide treatment effects on EAE induced in DA rats with homogenate injection. The animals induced with homogenate injection were treated with pCav3 (500 mcg/kg) once a day, while the control group had non-treated EAE. The animals were terminated on the 11th day after EAE was induced and underwent spinal cord histology examination. Microscopic examination of the H&E stained slices revealed that the changes in the spinal cord coincided with the disease scores in the animals with EAE, those treated with pCav3, and non-treated EAE (Figure 5). Drastic changes were observed in the spinal cords (lumbalic zone) of the animals with non-treated EAE compared to EAE treated with pCav3 peptide. The neuronal tissue of the animals with non-treated EAE was infiltrated with inflammatory cells, forming characteristic foci (Figure 6A). Generally, inflammatory cell foci, formed in an infiltrated spinal cord during EAE, can be stained with a standard H&E procedure, given the rather unique properties of acidic eosin versus basic hematoxylin. Infiltrated inflammatory cells containing nucleic acids are visualized with the basic stain versus the myelin-rich background as a result of staining with an acidic compound. The detection of cells infiltrating neuronal tissue are linked to disease symptoms’ severity. H&E staining is a typical procedure to monitor EAE status, allowing for the visualization of a particular effect (cells infiltrated in the spinal cord) resulting from the EAE treatment.

### 3.5. Results of CIA Treatment with Peptide

**Figure 7:**
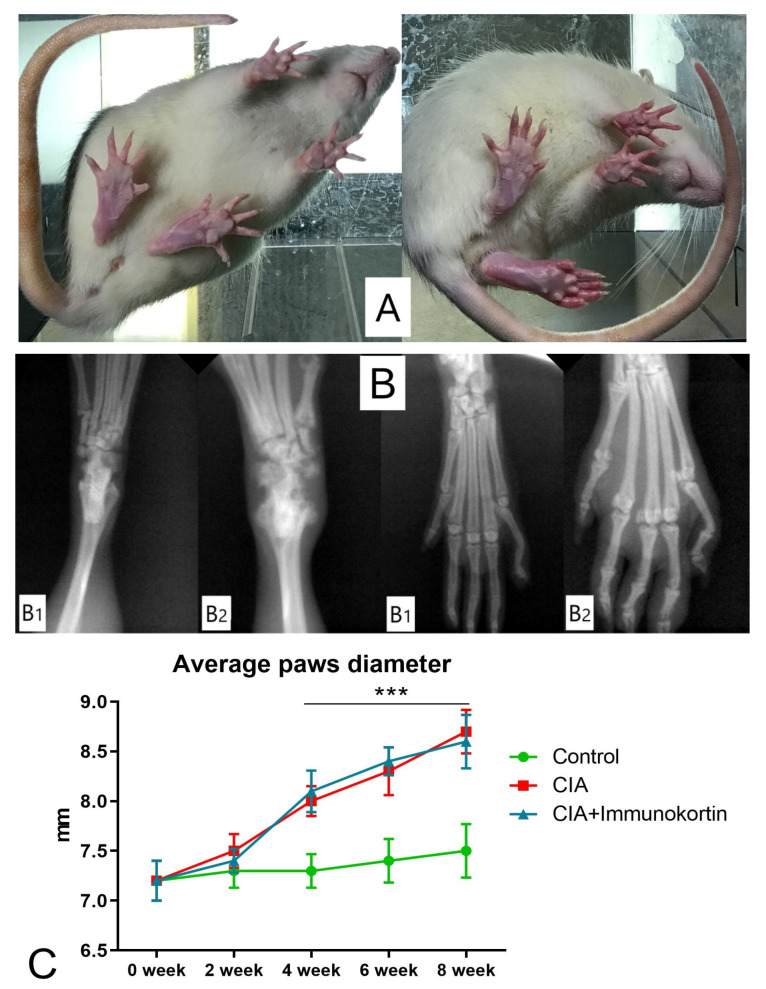
Collagen II-induced arthritis (CIA): (**A**) Animals, injected with saline (**left**) or collagen II (**right**); (**B**) X-ray pictures of hind paws of animals, injected with saline (**B1**) or collagen II (**B2**); (**C**) hind paw diameters (averaged) were measured at 0, 2, 4, 6, and 8 weeks of study. Immunocortin was injected daily from the 4th to 7th week after animals were immunized with collagen II (the treatment lasted for 28 days). Control: group of intact animals; CIA: group of animals with non-treated CIA; CIA+immunocortin: CIA treated with immunocortin, injected once a day (500 mcg/kg). *** Differences between CIA groups and control were confirmed by Kruskal–Wallis test, *p* < 0.001. Difference between CIA+immunocortin and CIA groups was not detected, according to one-way ANOVA (*p* < 0.05).

The averaged diameter of the hind paws in each group of animals was used to assess the CIA symptoms. Larger diameters of paw were detected in animals with CIA at 4, 6, and 8 weeks of the experiment compared to the control (Figure 7). We found no credible difference between the paw diameters of the rats in the CIA-treated and non-treated groups of animals at 4, 6, and 8 weeks of the experiment (Figure 7).

### 3.6. Disability Test Results

**Figure 8:**
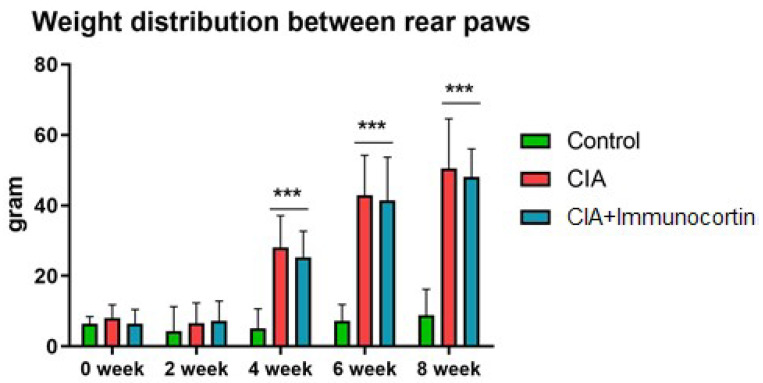
Distribution of averaged body weight put on affected paw was evaluated with in-capacitance tester on 0, 2, 4, 6 and 8 weeks after CIA initiation. Peptide immunocortin was injected once a day, starting on 4-th week, after CIA was induced. Control: intact group of animals; CIA: animals immunized with collagen II; CIA+immunocortin: animals with CIA treated with immunocortin injected once a day (500 mcg/kg). *** Kruskal-Wallis and multiple comparison Dann tests confirmed data difference relative to Control (*p* < 0.001).

Joint damage was detected as a distinctive anatomical displacement, estimated with a disability test, which assessed the weight distribution between the damaged and intact hind paws. The control animals showed an equal distribution of weight between the rear paws. The animals with CIA demonstrated a significantly lower weight on the affected paw as detected at 4, 6, and 8 weeks, although the injections of immunocortin peptide resulted in no credible change in the paw diameters compared to the animals in the non-treated CIA group (Figure 8).

## 4. Discussion

We investigated synthetic peptide pCav3 corresponding to the C-terminal part of caveolin-3 in vitro and in vivo. We found that pCav3 injected into DA rats daily was effective against the development and acute phases of EAE—a model of autoimmune disorders, such as MS in humans. Peptides from caveolin-3 and immunocortin demonstrated an ability to interfere with propagating ConA-stimulated DLN lymphocytes extracted from DA rats on the ninth day after they were primed with homogenate injection. Given the known ability of immunocortin to reduce EAE scores in DA rats, we tried but failed to reveal the least measurable effect of immunocortin on CIA induced in SD rats. That experiment was set up by following the OECD recommendations, which claim that DA rats have too-complex immunological responses and are not recommended by the OECD to model CIA in preclinical studies. Following this recommendation, we had no choice but to use a different rat strain—SD rats, which is recommended by the OECD to model RA in the form of CIA for preclinical trials. Interestingly, this line of rats was previously genotyped with an f-type of the MHC RT1 locus that is different from DA rats. Overall, CIA has been established with collagen II immunization in Wistar, Sprague–Dawley, and Wistar–Lewis rats, mice, and some other primates [25,26]. In preclinical trials, CIA has been used for modeling rheumatoid arthritis (RA) in rats, since CIA and RA have the same main immune-pathological features. Nevertheless, susceptibility to CIA established in different kinds of animals is linked to the MHC type: CIA in mice is heavily directed by the MHC type, while CIA in rats is less sensitive to the genetic base. Despite the variety of rat strains bearing different MHC types that are susceptible to collagen II-induced arthritis, the incidence rate, magnitude of symptoms, and CIA severity could be different. This also includes antibodies and the scope of the T-cell reactive epitope specificity [28,29,30].

It is accepted that naïve T cells interact with dendritic cells, or antigen-presenting cells (APCs) are set to engage T-cell activation. During this process, the antigen is upregulated by APCs for processing, so the antigen fragments are then exposed to the APC cell surface in the form of an antigen-MHC-I complex (Ag-MHC-I). Complimentary structures of T-cell antigen receptors (TCRs) can also recognize Ag-MHC structures, forming an Ag-MHC-II/TCR triple immune complex (Signal-1). Accomplishing T-cell activation requires Signal-2 to happen, which is featured by stimulatory molecular complexes formed in the APC/T cell contact areas [36]. Inflammatory-like responses can be developed as a result of Signal-2 expansion via co-stimulatory molecule interaction during T-cell activation [37,38]. Both of the signals (Signal-1 and Signal-2) shape the so-called structure of immunological synapse (IS) [39], which involves the lateral translocations of Signal-1- and Signal-2-related complex structures, as demonstrated previously on MHC-II/p/TCR and ICAM-1/LFA-1 complexes [40,41]. Data point to the MHC-II/p/TCR complexes of Signal-1, situated in the periphery of Signal-2-related structures [39]. Yet, they can swiftly move from the peripheral area to the center to form another characteristic structure of the central supramolecular activation complex (cSMAC), which marks T-cell activation, influencing immune reaction development.

MS and RA have some factors in common, since RA is also spread over the world and affects roughly 1% of the human population [42]. Therefore, in clinics, MS and RA share some general treatments for what are considered common problems of autoimmune disorders with unknown exact causes [43]. The recognition of genetic predisposition to developing autoimmune disease is one of the major factors that should be taken into consideration, given that the prevalence of disease may vary in different populations. For instance, the native tribes of North America are predisposed to MS much less compared to RA [44]. It was noticed that females are more susceptible to RA than males (with a ratio above 2:1), which indicates a possible role of hormones in RA pathogenesis [45].

Since EAE in DA rats was proven as a common model of human auto-immune demyelinizing neuropathy, the results of EAE treatment with pCav3 support this peptide as a candidate for drugs to be evaluated as a possible treatment for auto-immune diseases, including Guillain–Barre syndrome, RA, or MS in humans. Symptoms of autoimmune response are shaped by autoreactive T cells, activated in response to a specific antigen, so the overall outcome is driven by the balance between coexisting but opposite processes: the propagation of antigen-specific T cells (AST) while eliminating AST cells. Some compositions are known to affect both EAE and CIA at the same time [46]. Based on this information, this rationale could be used for testing the ability of immunocortin-class peptides to affect the scores of not only EAE but also CIA, considering the peptide activity based either on a shifting immune cell balance or resulting from a genuine antigen restriction process. It was shown previously that impaired recovery from EAE could result from the used apoptosis inhibitors responsible for suppressed inflammatory cell apoptosis [47]. At the same time, the induction of autoreactive T-cell apoptosis diminished the EAE symptoms [48,49]. Furthermore, a study reported that floral extract kirenol was capable of inhibiting mature antigen-specific T-cell survival; namely, it could raise the number of naive T cells by lowering the number of effector/memory T cells and then inducing the cell apoptosis of lymphocytes extracted from the drained lymph nodes of kirenol-treated animals [50].

The use of immune-modulating peptides for the treatment of autoimmune disorders minimizes the threat of forceful depletion of the immune system from the main immune cell subpopulations. This is contrary to the common side effects observed as a result of contemporary drugs used to treat MS in clinics, including recombinant cytokines or humanized antibodies (hAb) developed against cell-surface antigens. It is noteworthy to point out that peptide-based drugs could be safer by helping to avoid leucopenia development. Indeed, immune-modulating peptides could minimize the magnitude and rate of side effects due to rapid turnover and limited cell penetration ability. Complex preclinical and toxicological trials are further required to fully assess the prospects of pCav3 and immunocortin for use as a novel medication to treat autoimmune disorders.

## 5. Conclusions

A novel peptide pCav3 was located in the C-terminus of the Cav3 region and found to be highly homologous to the immune-modulator peptide immunocortin.

Both the structures of pCav3 and immunocortin, used for molecular docking, provided the peptide ligand binding energy necessary after peptide was placed within the triple auto-immune MHC/P/TCR complex.

Synthetic peptides of pCav3 and immunocortin demonstrated similar activity on EAE in DA rats immunized with horde_homogenate.

In addition, the synthetic peptides of pCav3 and immunocortin demonstrated similar anti-proliferative activity on DLN lymphocytes extracted from the DA rats primed with horde homogenate injection.

Immunocortin failed to influence on the CIA scores in SD rats.

Since immunocortin performed differently on two distinct models of autoimmune diseases, we assume that the OECD recommendations for avoiding DA rats to model CIA in favor of SD rats require further consideration. The results could be influenced by the fact that DA and SD rats aregenetically different by their genomic sequences in the MHC RT1 loci. The RT1 locus directs MHC protein expression, providing antigen recognition and structure restriction, which greatly shape the immune response seen during EAE and CIA modeled in rats.

It was concluded that immunocortin could interact differently with MHC proteins expressed by distinct MHC RT1 loci.

Further considerations are required for using DA rats for combined or comparative studies of the CIA and EAE treated with the same drug.

## Figures and Tables

**Table 1:** Ligand binding energy (Δ*E_bind_*) was calculated by using the free energies of the triple complex MHC/p/TCR and separate complex structure components—isolated ligand and complex with no ligand.

Ligand Source	Ligand Structure *	Δ*E_bind_*, kJ/mol
BMP	RGGA**S**Q**YR**PSQ	403
pCav3 (136–146 a.a.)	LGQV**CSSIKV**V	506
Immunocortin	VKKPG**CSSVKV**	496

* Identical or highly similar amino acid residues are labeled with red.

**Table 2:** Cell DNA containment in DLN lymphocyte distribution. DLN lymphocytes were extracted from DA rats primed with homogenate injection and then cultivated with pCav3 peptide (20 mcg/mL) or ConA (5 mcg/mL) for 48 h, followed by cell cytofluorimetric analysis.

Cell Cycle Phase	Control (%)	pCav3 (%)	pCav3 + ConA (%) ^1,2^	ConA ^1,2^
G1 ^3^	59 ± 2.5	59.3 ± 2.4	56.3 ± 2.0	50 ± 2.1
G2 + M ^3^	12.2 ± 0.5	12.1 ± 0.6	11.7 ± 0.6	8.5 ± 0.3
S ^3^	23.9 ± 1.2	23.5 ± 1.1	27.4 ± 1.4	37.8 ± 1.7

^1^ *p* = 0.037 relative to control data set (Mann–Whitney test). ^2^ *p* = 0.038 relative to control data set (Kholmogorov–Smirnov, KS test). ^3^ *p* = 0.038 pCav3 + ConA data compared to ConA only (Kholmogorov–Smirnov, KS test).

**Table 3:** Cumulative EAE scores in groups treated with peptides on days 11–28, including the control group of animals with non-treated EAE.

Groups	EAE/Group Integral Scores							
	11 Day	12 Day	13 Day	14 Day	15 Day	16 Day	17 Day	18 Day
Non-treated EAE	20	23.5	24	22.5	18.5	14	4	-
* pCav3/EAE	6	6	10	14	12.5	8	3	-
* Immunocortin/EAE	4	7	13	9	10	9	3	-

* Differences between EAE treated with peptide and control animals (non-treated EAE) were confirmed with Kholmogorov–Smirnov and Kruskal–Wallis tests (*p* < 0.05).

## Data Availability

The data supporting the reported results are available from D.A.V and V.A.P. upon request.
